# Development of carboxymethyl cellulose-graphene oxide biobased composite for the removal of methylene blue cationic dye model contaminate from wastewater

**DOI:** 10.1038/s41598-023-41431-8

**Published:** 2023-08-31

**Authors:** Eman N. Mohamed, Ahmed I. Abd-Elhamid, Ali A. El-Bardan, Hesham M. A. Soliman, Mohamed S. Mohy-Eldin

**Affiliations:** 1 Department of Chemistry, Faculty of Science, Alexandria University, P.O.Box 426, Alexandria, 21321 Egypt; 2 Advanced Technology and New Materials Research Institute (ATNMRI), City of Scientific Research and Technological Applications (SRTA-City), New Borg Al Arab, 21934 Alexandria Egypt; 3 Polymer Materials Research Department, Advanced Technology and New Materials Research Institute (ATNMRI), City of Scientific Research and Technological Applications (SRTA-City), New Borg Al Arab, 21934 Alexandria Egypt

**Keywords:** Environmental social sciences, Materials science

## Abstract

Utilizing Glutaraldehyde crosslinked sodium carboxymethyl cellulose (CMC-GA) hydrogel and its nanographene oxide composite (CMC-GA-GOx), an effective carboxymethyl cellulose-graphene oxide biobased composites adsorbent was developed for the adsorption removal of methylene blue (MB) cationic dye contaminate from industrial wastewater. The CMC-GA-GOx composites developed were characterized using FTIR, RAMAN, TGA, SEM, and EDX analysis instruments. Through batch experiments, several variables affecting the removal of MB dye, including the biocomposites GO:CMC composition, adsorption time, pH and temperature, initial MB concentration, adsorbent dosage, and NaCl concentration, were investigated under different conditions. The maximum dye removal percentages ranged between 93 and 98%. They were obtained using biocomposites CMC-GA-GO_102_ with 20% GO weight percent, adsorption time 25 min, adsorption temperature 25 °C, MB concentrations 10–30 ppm, adsorption pH 7.0, and 0.2 g adsorbent dose. The experimental data of the adsorption process suit the Langmuir isotherm more closely with a maximal monolayer adsorption capacity of 76.92 mg/g. The adsorption process followed the kinetic model of pseudo-second order. The removal of MB was exothermic and spontaneous from a thermodynamic standpoint. In addition, thermodynamic results demonstrated that adsorption operates most effectively at low temperatures. Finally, the reusability of the developed CMC-GA-GO_102_ has been proved through 10 successive cycles where only 14% of the MB dye removal percentage was lost. These results suggest that the developed CMC-GA-GO_102_ composite may be an inexpensive and reusable adsorbent for removing organic cationic dyes from industrial wastewater.

## Introduction

Water is one of the essential elements for all life on earth, and it has been a boon to human civilization since ancient times. However, water is one of the most challenging environmental issues, and supplying safe, affordable drinking water and clean water is a global challenge. The swiftly deteriorating environmental contamination of natural resources is a contemporary issue that must be addressed immediately if the earth and its inhabitants are to endure for future generations^1,2^. Large quantities of water have become contaminated in recent years due to the rapid development of various technological fields, leading to increased environmental disturbances and severe pollution issues^3^. In general, water pollution can occur in a variety of ways; however, a significant amount of water pollution is caused by the contamination with dyes as a result of numerous industrial effluents from paper, cosmetics, leather, pigments, petroleum, textiles, plastic, refineries, printing, pharmaceutical, and food processing industries^4–6^.

According to their dissociation behavior in aqueous solutions, dyes can be divided into three categories: (a) anionic (acid, direct, and reactive dyes) with a negative charge primarily owing to the (SO_3_) group, (b) cationic (basic dyes) with a protonated amine group, and (c) nonionic (disperse dyes)^7^. Cationic or anionic azo dyes contain one or more azoic bonds (N=N). In addition to its resistance to light, heat, and aerobic digestion, it can cause acute diseases such as genetic mutation, allergic reactions, vomiting, and cyanosis^8^, resulting in environmental pollution and attendant health concerns for all living organisms, including humans, animals, and plants^9–11^. These dyes adversely affect human health due to their chemical stability and nonbiodegradability. Previous research has demonstrated that cationic dyes are highly toxic and more harmful than anionic dyes because they readily interact with cytoplasm^12–15^.

Methylene blue (C16H18N3ClS) is both a thiazine and cationic dye. MB is predominantly used as a colorant in the textile industry, particularly in wool, silk, and cotton dyeing. It is widely used in industrial processes, including biomedical pigments for cell staining, paper printing and pulp, rubber, plastics, leather, cosmetics, and textile coloring. Due to its toxicity, carcinogenicity, mutagenicity, and nonbiodegradability, it is considered the most harmful organic pollutant in the water environment. It can cause nausea, mental confusion, vomiting, eye burns, mental confusion, cyanosis, high blood pressure, tachycardia, abdominal pain, skin irritation, methemoglobinemia, red blood cell breakdown, dyspnea, profuse sweating, gastrointestinal irritation, allergic reaction, diarrhea in humans. Additionally, its inhalation causes shortness of breath after brief exposure^13,16–18^.

Dyes-contaminated wastewater is more challenging to manage due to its complex chemical composition, including inorganic filler, organic solvent, polymer, and toxic dyes. Therefore, removing toxic dyes from the effluent is a pressing concern. Before discharging industrial effluent into water streams, removing these contaminants and reducing the concentration of these dyes is essential. Current dye-polluted wastewater treatment methods include adsorption, oxidation, coagulation, electrolysis, photocatalytic degradation, biodegradation, and liquid membrane separation. Due to the complex aromatic structures of dyes, which make them highly stable, conventional dye removal techniques are frequently ineffective. Adsorption is a process that researchers and industry are interested in because of its low cost, ease of regeneration, and availability of various adsorbents. It has recently received much attention due to its low cost, ease of regeneration, and availability of various adsorbents. It is now considered a simple, more accessible, economical, eco-friendly, and effective technology for removing various dyes^19–21^.

Numerous organic and inorganic adsorbents, such as ion exchange resins, fly ash, carbon-based materials, mesoporous silica, polysaccharides, zeolites, clays, and hydrogel nanocomposites, have been studied for the removal of dyes. However, they have disadvantages such as low adsorption capacities, low selectivity, and high cost. Therefore, efforts are being made to develop versatile, cost-effective, and efficient adsorbents for water purification applications^22,23^.

For wastewater remediation, a natural polymer such as polysaccharide has received much attention for environmental reasons. Due to their economic viability and environmental significance, polysaccharides such as starch, chitosan, alginate, and carboxymethyl cellulose have been extensively used as efficient adsorbents in wastewater treatment fields due to their economic viability and environmental significance^24^. In addition, they are remarkably inexpensive, abundant, and biocompatible.

Carboxymethyl cellulose (CMC) is an anionic linear polysaccharide derived from cellulose that contains (–CH_2_–COOH) groups linked to the (OH) groups of glucopyranose monomers, which form the cellulose backbone, and a high number of active functional groups including hydroxyl and carboxyl groups that serve as desirable active sites during the adsorption process. It is the most popular and environmentally preferred polymer that is water-soluble. It typically contains significant amounts of active functional groups, such as hydroxyl and carboxyl, which are desirable active sites during adsorption. CMC is also an inexpensive, non-toxic, biodegradable, and renewable polymer^25–27^. However, CMC has weak mechanical properties and a low adsorption capacity despite its extensive use in wastewater treatment. Several physical and chemical modification techniques for CMC, such as composite formation and grafting, have been devised to overcome these drawbacks^28,29^.

On the other hand, graphene oxide (GO) is produced by the thermal oxidation of the two-dimensional carbon allotrope graphene, which is a highly motivating material. Various oxygen functional groups (–OH, –O–, –COOH, diol, etc.) are located on the surface and edges of graphene oxide, leading to its widespread use in adsorption for wastewater treatment. These properties include a large specific surface area, low toxicity, high ionic exchange, biocompatibility, and high hydrophilicity. So, graphene oxide (GO) is presently used as an adsorbent material for various pollutants in wastewater, including heavy metals and dyes. However, it is challenging to separate GO after adsorption, so several experiments have been conducted to combine GO and CMC to form an effective structure of GO/CMC-based composites for future applications^30–32^.

In response to this interest, a study was conducted to develop an effective carboxymethyl cellulose-graphene oxide biobased composites adsorbent for removing cationic MB dye using Glutaraldehyde-bonded sodium carboxymethyl cellulose (CMC-GA) hydrogel and graphene oxide (CMC-GA-GO). This study investigates the merging of different approaches for modifying carboxymethyl cellulose with GO to enhance its sorption of MB dye. These approaches include the followings:

1. Chemical crosslinking of the CMC to create a three-dimensional porous structure with improved mechanical properties and surface area,
2. Physical crosslinking through the free drying technique creates additional porosity and internal surface area,
3. It induces additional adsorption sites by mixing GO particles with additional surface area.

Finally, we have obtained novel physical and chemical crosslinked CMC-GA-GOx composites with high porosity, surface area, and appreciated numbers of active sites for removing cationic dye from aqueous solution. The CMC-GA-GOx was characterized using FTIR, RAMAN, XRD, SEM, TEM, EDX, and TGA. The contact time, initial dye concentrations, adsorbent dosage, pH, temperature, and other adsorption batch conditions were investigated. In addition, isotherms, kinetics, and adsorption thermodynamics parameters were determined.

## Materials and methods

### Materials

Sodium carboxymethyl cellulose (CMC) was obtained from Across (99%), graphite (200 mesh, 99.99%) was purchased from Alpha Aesar), sulfuric acid (95–97%) was obtained from Riedel deHaen). Potassium permanganate (99%, Long live), and glutaraldehyde (GA; 25%) were acquired from Sigma Aldrich. Hydrochloric acid (HCl; 30%) was obtained from El Salam for Chemical Industries, Egypt. Sodium hydroxide, hydrogen peroxide (H_2_O_2_; 36%) were obtained from Pharaohs Trading and Import, Egypt. Methylene blue (MB) was used (99%, Sigma-Aldrich). All chemicals were used exactly as they were delivered.and all other prepared solutions were made with distilled water (2108, GLF, Germany).

### Preparation of GO

Our previous research mentioned that graphene oxide was synthesized using the Hummers' method^33^. Briefly, 3.0 g of graphite powder was added to a 70 mL solution of concentrated sulfuric acid in a 150 mL glass beaker while agitating for 10 min. This suspension was kept at 20 °C in a water bath while 9.0 g KMnO_4_ was steadily added. Afterward, 3.0 g of potassium persulfate was added progressively. Afterward, the mixture's temperature was increased to 40 °C with vigorous stirring for approximately half an hour (Slurry A). Next, the Slurry (A) was mixed with 150 mL of water and agitated at 95 °C for 15 min. Then, 500 mL of water was added to the final slurry (Slurry B), followed by the gradual addition of 15 mL of 30% H_2_O_2_, which caused the mixture's color to change from dark brown to yellow. Finally, the suspension was filtered and rinsed with 1:10 HCl aqueous solutions (250 mL) followed by 250 mL bi-distilled water and dried at 35 °C for 99 h to remove metal ions. The substance obtained (GO) was used for further research.

### Construction of the CMC-GA

0.3 g CMC powder was dissolved in 30 mL distilled water at room temperature under constant stirring for 24 h. Next, HCl acidified 0.25 mL glutaraldehyde (25%) was added to the solution as a cross-linker. The mixture was stirred at 300 rpm for 60 min at 30 °C before being lyophilized dried for 48 h using Christ-freeze dryer Alpha 1-4 LD-2.

### Synthesis of CMC-GA-GOx composites

0.3 g of CMC was dissolved in 30 mL of distilled water at ambient temperature for three hours with constant stirring. To ensure a uniform distribution of graphene oxide (GO), 2, 4, 6, and 10 mL of graphene oxide suspension (1.5% w/w) were added to the CMC solution and agitated continuously for 24 h (to ensure complete dissolution). Then 0.25 mL of HCl-acidified 25% glutaraldehyde was added. At 30 °C, the mixture was stirred for an additional 60 min. The CMC-GA-GOx composites (x = 101, 102, 103, and 105) were obtained (Table 1), and the resulting composites were desiccated using a lyophilizer under the same conditions used to dry the CMC-GA.

**Table 1:** The compositions of different CMC-GA-GOx composites.

Sample code	GA (ml)	CMC (g)	GO (g)	GO:CMC ratio (W%)
CMC-GA	0.25	0.3	0.00	0.0
CMC-GA-GO_101_	0.25	0.3	0.03	10
CMC-GA-GO_102_	0.25	0.3	0.06	20
CMC-GA-GO_103_	0.25	0.3	0.09	30
CMC-GA-GO_105_	0.25	0.3	0.15	50

### Characteristics

Changes in the morphology of CMC-GA and CMC-GA-GO_x_ composites were examined using a scanning electron microscope (JEOL GSM-6610LV, Tokyo, Japan) at acceleration voltage of 15–20 kV. Specimen surfaces were coated with a thin layer of gold before observation. Elemental analysis was carried out using the EDX unit. SEM images were obtained at different magnifications. EDX-SEM analysis is conducted on GO, CMC, CMC-GA, CMC-GA-GO_102_, and CMC-GA-GO_102_-MB to determine the elemental composition of the investigated materials and confirm dye adsorption by the investigated composites.

Raman spectroscopy was used to record the Raman spectra of the sample in order to identify the chemical structure changes (BRUKER, OPTIK GMBH, Senterra, Germany). Thermogravimetric analysis (Shimadzu Thermal Gravimetric Analysis (TGA) 50, Tokyo, Japan) determined the samples' thermal stability under the following operational conditions: 10 °C min^−1^ heating rate in a dynamic nitrogen atmosphere as an inert gas between room temperature and 800 °C. The Fourier Transmission Infrared Spectroscopy (FT-IR) (8400 s, Shimadzu, Kyoto, Japan), with a range of 400 to 4000 cm^−1^, was used to detect functional group modifications. The KBr disc method was employed to detect the IR spectra of each sample under investigation.

### Adsorption research

In order to assess the adsorption procedure, bulk adsorption experiments were conducted. The desired concentration was attained by dilution, preparing a stock solution of 1000 ppm MB dye by dissolving 0.1 g of each dye in 100 mL of distilled water. In 50 mL of MB dye solution (30 mg/L), 0.02 g of CMC-GA-GO_102_ composite was accurately inserted. The pH of the adsorption medium was adjusted between 2 and 11 using 0.1 M solutions of both HCl and NaOH for contact time (0–120 min). Experiments were conducted at a constant rate of agitation (300 rpm) and a temperature of 25 °C. After it has reached equilibrium, separate the adsorbent from the dye solution by centrifuging it at 3000 rpm for 10 min. At an absorbance wavelength of 662 nm, a UV–Vis spectrophotometer was utilized to measure the residual concentration of MB dye.

Mathematical models of equilibrium and kinetics were developed utilizing the collected data. Without adjusting the pH, 0.02 g of CMC-GA-GO_102_ was added under constant agitation to 100 mL Erlenmeyer flasks containing 50 mL of dye solution at the specified concentration (30 mg L^−1^ of MB). The isotherms, thermodynamics, and kinetics of adsorption were investigated. The MB dye residual concentration in the aqueous solution was calculated using the initial dye concentration and absorbance measurements before and after adsorptions. The removal percentage (R%) and adsorption capacities of MB at time t were determined using the following equations:

1 $$R \left(\%\right)=\frac{{C}_{0- {C}_{t}}}{{C}_{0}}\times 100$$

2 $$\mathrm{qt }= \frac{({c}_{0}-{c}_{t})v}{1000w}$$

C_0_ is the initial dye concentration (mg L^−1^), C_t_ is the dye concentration at different time intervals (mg L^−1^), V is the volume of dye solution (L), and W is the mass of adsorbent (g). R (%) represents the removal efficiency of MB dye using the adsorbents, and q_t_ is the adsorption capacity (mg/g).

### Adsorption reusability

0.02 g of CMC-GA-GO_102_ adsorbent mixed with 50 mL of 10 ppm MB solution of pH 7.0 for 25 min at room temperature and agitated at 300 rpm. After completion of the adsorption time, the adsorbent was separated from the dye solution by centrifuging it at 3000 rpm for 10 min. The dye removal percentage was measured as mentioned previously. The CMC-GA-GO_102-_MB adsorbent was mixed with 100 mL NaCl solution dissolved at DW of 3% concentration at room temperature using a mechanical stirrer at 250 rpm for 60 min to desorbs MB molecules, then separated as mentioned previously and washed three successive times with distilled water before separating and used in further adsorption experiments (10 cycles) under the same MB adsorption conditions.

## Results and discussion

### Materials characterization

#### FTIR analysis

**Figure 1:**
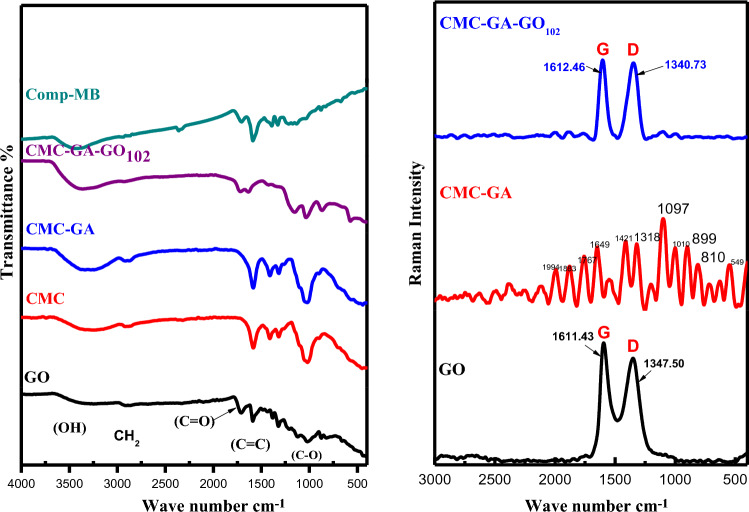
(**A**) FTIR spectra of GO, CMC, CMC-GA, CMC-GA-GO_102_, and CMC-GA-GO_102_-MB. (**B**) Raman spectroscopy of GO, CMC-GA, and CMC-GA-GO_102_.

The chemical structure of GO, CMC, CMC-GA, CMC-GA-GO_102_, and CMC-GA-GO_102_-MB was confirmed by Fourier transform infrared spectroscopy (Fig. 1A). The FT-IR spectrum of GO reveals characteristic peaks at 3425, 2912, 1710, 1589, 1484, 1209, 1021, and 1383 cm^−1^, corresponding to OH, C–H, C=O, C=C (aromatic ring), O–C=O, epoxy C–O–C, alkoxy C–O, and carboxyl (C–O), respectively. That indicates that the GO surface is rich in oxygen-containing functional groups. The principal peak at 1589 cm^−1^ corresponds to C=O in the COOH unit of GO. These firm peaks of stretching vibration of oxygen-containing functional groups indicate the presence of many oxygen-containing functional groups on the surface of GO^11,34,35^. In contrast, for the CMC-GA-GO_102_ Composite, the characteristic C=O band of GO is visible but not readily apparent, indicating the successful incorporation of GO. This peak's lack of visibility could be attributed to its low GO concentration^36^.

Pure CMCNa bands at 1583 cm^−1^ (*vas*COO) and 1412 cm^−1^ (*vs*COO) corresponds to the antisymmetric and symmetric stretching of carboxylate groups, respectively, and the O–H stretching band appeared at 3244 cm^−1^. The CH stretching frequency is 2920 cm^−1^. At 1319 cm^−1^, skeletal vibrations are also observed. The bending frequency of CC is 1019 cm^−1^^37,38^.

CMC-GA and CMC-GA-GO_102_ have comparable characteristic peaks in their FT-IR spectra. The OH band was stretched at 3275 and 3362 cm^−1^, and the CH band at 2916 and 2929 cm^−1^ for CMC-GA and CMC-GA-GO_102_, respectively^37^.On the spectrum of CMC-GA, two distinct peaks are observed at 3275 and 1022 cm^−1^, corresponding to –OH and C–O–C stretching vibrations, respectively. Despite these two peaks in the CMC-GA-GO_102_ composite, a clear red shift of the –OH peak from 3275 to 3362 cm^−1^ can be observed due to the presence of GO in the CMC-GA matrix. It indicates a robust hydrogen bonding interaction between CMC and GO^19^. The characteristic band of C=O of GO cannot be observed in CMC or CMC-GA. However, after incorporating GO into the CMC-GA-GO_102_ composite, the absorption band at 1719 cm^−1^ can still be observed (Fig. 1), indicating that the CMCNa cross-linking was successful. Moreover, at approximately 1636 cm^−1^, the carbonyl stretch of the carboxylic groups of GO overlapped with the carboxylate groups of CMCNa, indicating a strong interaction between the carboxyl groups of GO and the hydroxyl groups of CMCNa^39^. The comparison of FTIR spectra reveals that the CMC-GA-GO_102_ composite contains an abundance of hydrophilic groups, including hydroxyl, carboxyl, and epoxy groups, which all have excellent chemical activity and aid in the adsorption of cationic dye^40^.

In addition, by combining CMC-GA-GO_102_ with the MB dye molecules, the FTIR- spectra will exhibit dye-specific peaks. After the adsorption processes of dye species, shifts, disappearances, the emergence of new bands, and alterations in peaks were observed. The peak at 3362 cm^−1^ shifted to 3434 cm^−1^, the peak at 1636 cm^−1^ shifted to 1588 cm^−1^, the peak at 1319 cm^−1^ shifted to 1391 cm^−1^, and the peak at 1268 cm^−1^ shifted to 1215 cm^−1^.

After surface adsorption of MB dye, the intensity and location of peaks in the structure of CMC-GA-GO_102_ nanocomposite changed. It signifies a reaction between the adsorbent and the contaminant dye. In addition, after MB dye adsorption, novel bands at 3434 cm^−1^ were observed, which were attributed to the presence of MB. After MB dye adsorption, the range and intensity of peaks in CMC-GA-GO_102_ nanocomposite change very little. These minute differences may indicate the existence of physical mechanisms (such as van der Waals forces) between the structure of the adsorbent and the pigment molecules.

The obtained data indicate that the CMC-GA-GO_102_ composite successfully eliminated MB dye. The minor shifts in the OH group suggest that hydroxyl-hydrogen atoms participated in the hydrogen bonding interaction between the composite and the dye^12,41^. In addition to ionic interactions between the -OH groups in the CMC-GA-GO_102_ structure, it is believed that numerous abrupt decreases are due to MB species, with the band shifting indicating that these functional groups interact with the MB molecules^42^.

#### Raman analysis

Raman spectroscopy can provide valuable information regarding GO structure, especially structural alterations in prepared samples. Figure 1B depicts the Raman spectra of GO, CMC-GA, and the CMC-GA-GO_102_ composite. GO's Raman spectrum displayed two distinct peaks at 1611 cm^−1^ and 1347 cm^−1^, corresponding to the well-known G and D bands^43^. The D band with sp3 carbon is caused by defects in graphene oxide and staging disorder, whereas the hexagonal pressure mode of graphite causes the G band.

The ratio of the G/D peaks within the GO spectrum corresponds to the ratio of the Sp2 hybridized carbon bonds crystalline defects (G-band) and functional groups present (D-band); this value is typically between 0.8 and 1.2 and is also an excellent indicator of the oxygen content; the lower the ratio, the fewer functional groups are present. In graphitic materials, the D-band to G-band intensity (ID/IG ratio) is commonly used to characterize the defect density. The greater the number of sp2 hybrid carbon atoms, which corresponds to a higher order degree of GO^44,45^, the lower the ID/IG ratio. In this investigation, the ID/IG ratio of graphene oxide was determined to be 0.87. 0.92 was the ID/IG ratio of the CMC-GA-GO_102_ nanocomposite. CMC-GA-GO_102_ has a higher ID/IG ratio than GO, indicating that the nanocomposite comprises nanosheets of ordered carbon. Moreover, because CMC-GA-GO_102_ nanocomposites are less dense than graphene oxide, the intensities of the two peaks in GO and CMC-GA-GO_102_ nanocomposites were diminished. Moreover, a higher ID/IG ratio of CMC-GA-GO_102_ in comparison to GO suggested that some of the oxygenated functional groups of functionalized CMC-GA-GO_102_ were involved in network formation, resulting in a decrease in their concentration and an increase in the value of ID/IG ratio^19^. CMC-GA-GO_102_'s Raman spectrum displays the corresponding characteristic peaks, such as the soft peaks of C–C stretching at 1269 cm^−1^ and the C=C symmetric stretching peak at 1424 cm^−1^. Due to interactions between GO and CMC-GA, the composite's Raman peaks undergo changes in intensity or position.

The FT-Raman spectral bands of CMCNa and hybrid materials are highly similar. The band at 2907 cm^−1^ is attributed to C–H stretching, the band at 1118 cm^−1^ to symmetric stretching of the C(1)–O–C(4) group and ring respiration, and the band at 918 cm^−1^ to bending C(5)C(6)–H and HC(6)O.

#### SEM analysis

**Figure 2:**
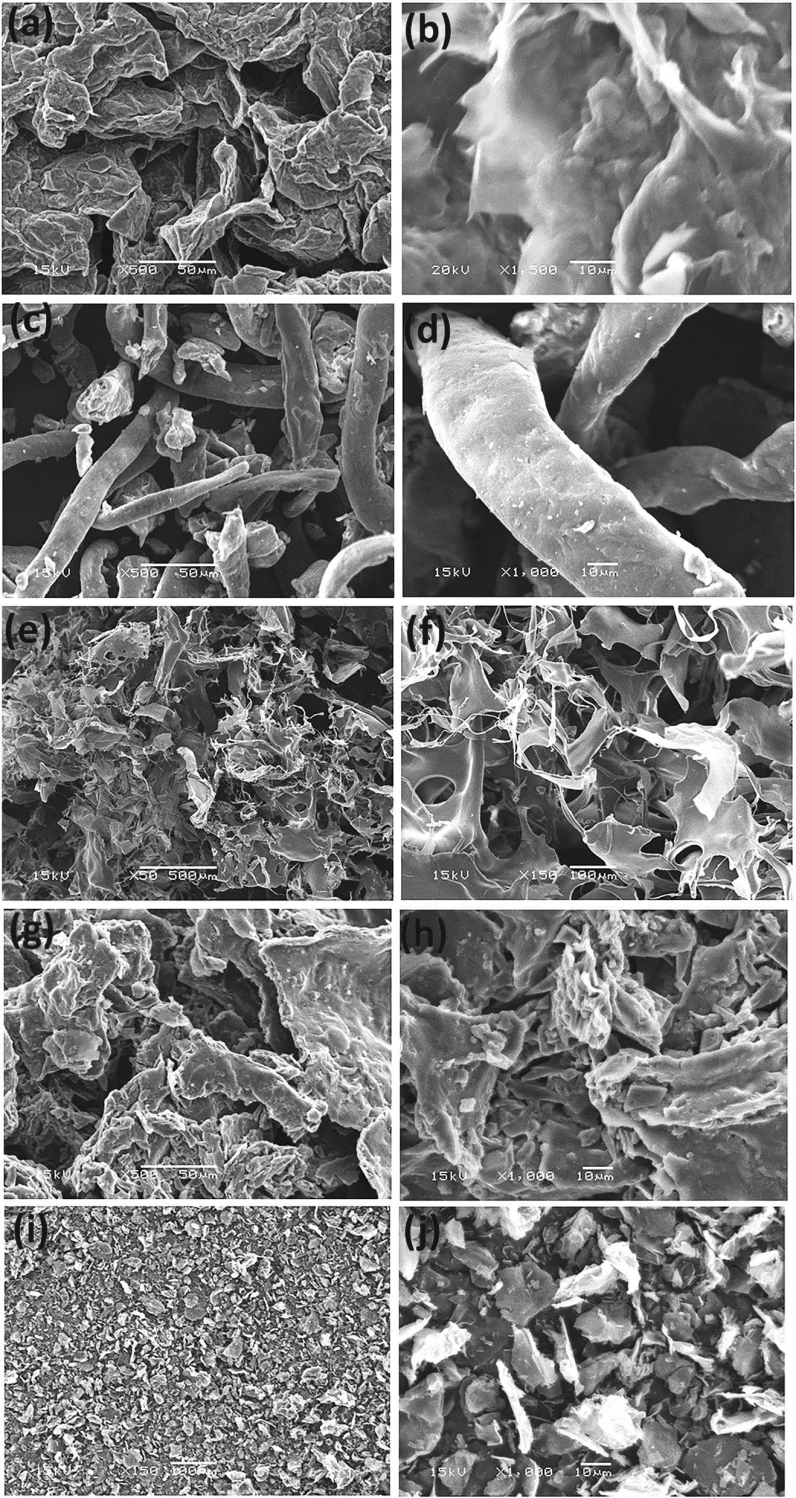
(**a**,**b**) SEM micrographs of GO. (**c**,**d**) SEM micrographs of pure CMC. (**e**,**f**) SEM micrographs of CMC-GA. (**g**,**h**) SEM micrographs of CMC-GA-GO_102_ composite before adsorption. (**i**,**j**) SEM micrographs of CMC-GA-GO_102_ composite after adsorption.

The surface morphology of an adsorbent is one of the most influential factors governing the adsorption process. Therefore, the composite's surface morphology and constituents were analyzed using scanning electron microscopy. Figure 2 depicts SEM images of (GO, CMC, CMC-GA, and the CMC-GA-GO_102_ generated composite sample) before and after dye adsorption. Figure 2 depicts GO sheets that resemble rounded pleats, a cloud, and a flat-surfaced sheet-like structure^46,47^. Conversely, CMC has a fibrous, woven surface structure^48,49^. The hydrogel's surface was virtually smooth and contained few pores. The SEM results for CMC-GA hydrogel and CMC-GA-GO_102_ nanocomposite hydrogel revealed a three-dimensional structure that is porous and interconnected.

Moreover, the composite SEM image revealed a rough structure, severe creases, and a uniform distribution of GO in the polymer matrices. In addition, the results demonstrated that adding GO and GA to the CMC chains increased the number and size of pores in the CMC-GA-GO_102_ structure. Consequently, it is anticipated that the CMC-GA-GO_102_ nanocomposite will have a higher removal capacity of MB from aqueous liquids than the CMC-GA^50–53^. After MB dye adsorption, the composite surface reflects no porous onto their surface.

#### EDX analysis

**Figure 3:**
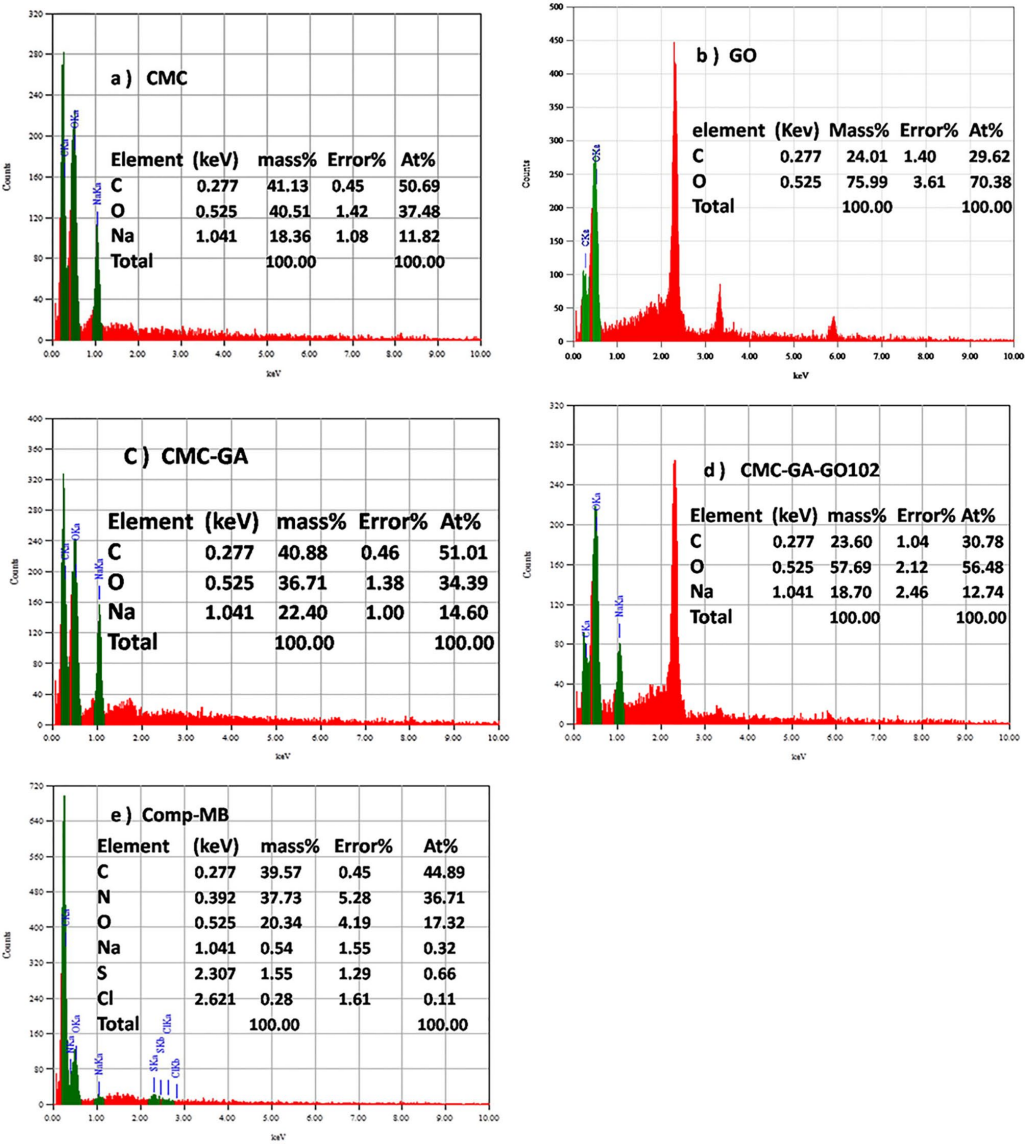
EDX analysis of pure CMC (**a**), GO (**b**), CMC-GA (**c**), CMC-GA-GO_102_ (**d**), and CMC-GA-GO_102_-MB composite (**e**)**.**

As demonstrated in Fig. 3, EDX-SEM analysis is conducted on GO, CMC, CMC-GA, CMC-GA-GO_102_, and CMC-GA-GO_102_-MB to determine the elemental composition of the investigated materials and confirm dye adsorption by the investigated composites. Before and after the MB adsorption procedure, the EDX analysis of the CMC-GA-GO_102_ composite was evaluated. CMC comprises C, O, and Na, as confirmed by Fig. 3A (elementary composition). The elemental analysis of GO reveals high concentrations of C and O, indicating that the synthesized GO is of good purity, while other three beaks at 2.3 keV, 3.4 keV, and finally 5.8 keV referred to remaining residues of S from sulfuric acid, K, and Mn from potassium permanganate used in the preparation method (Fig. 3B). Prior to the addition of GO, as shown in Fig. 3C, the atomic percentages of O, C, and Na in CMC-GA were 34.39%, 51.01%, and 14.60%, respectively. When GO is incorporated into the CMC-GA, the atomic percentages of O, C, and Na are observed to be 56.48%, 30.78%, and 12.78%, respectively in Fig. 3D. The decrease in carbon and sodium content and the increase in oxygen content indicate that GO incorporation has altered the chemical composition to include more oxide. However, the three beaks at 2.3 keV, 3.4 keV, and finally 5.8 keV referred to remaining residues of S from sulfuric acid, K, and Mn from potassium permanganate used in the preparation of GO (Fig. 3B) still show up with lower intensity. As shown in Fig. 3E, the CMC-GA-GO_102_-MB contains carbon, oxygen, nitrogen, sulfur, and chlorine after MB dye adsorption by CMC-GA-GO_102_, as well as a decrease in Na and O content, which may refer to the adsorbed dye.

#### TGA analysis

**Figure 4:**
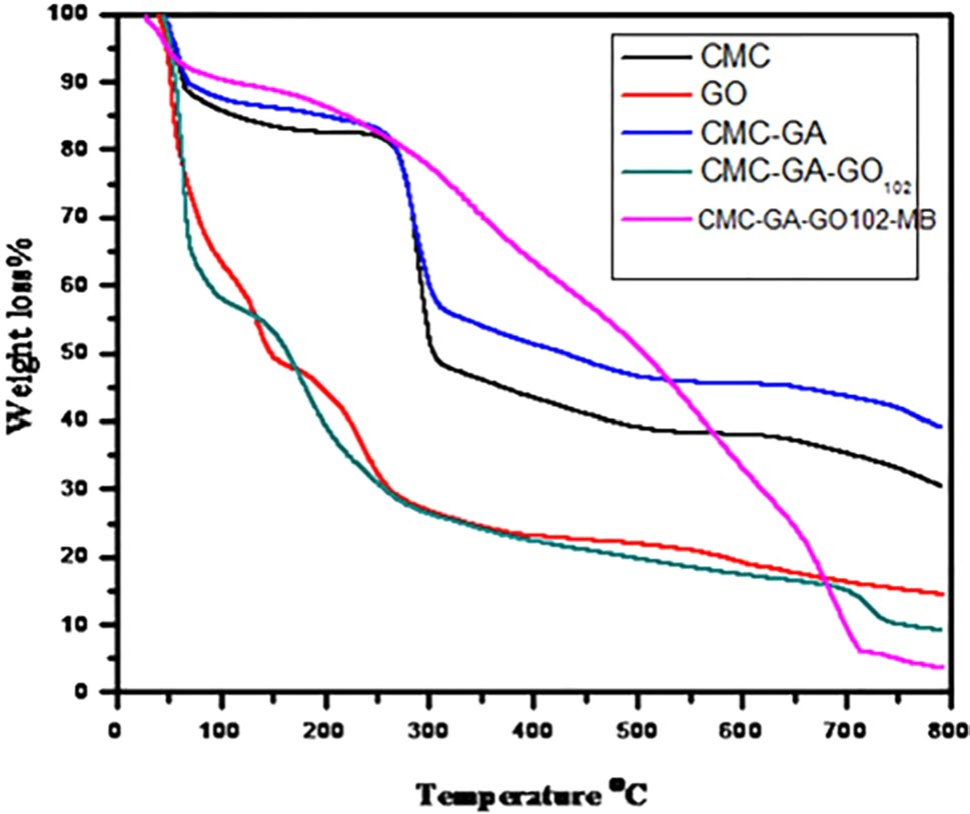
The thermogravimetric analysis (TGA) of GO, CMC, CMC-GA, CMC-GA-GO_102_, CMC-GA-GO_102_-MB complex.

Thermogravimetric analysis is an effective method for determining the temperature of weight loss, the rate of weight loss, and the weight of the sample after weight loss. The TGA analysis of CMC, GO, CMC-GA, CMC-GA-GO_102_, and CMC-GA-GO_102_-MB is depicted in Fig. 4. On the TGA curves, as depicted in Fig. 4, the weight loss of the samples could be broken down into several stages. The weight loss below 100 °C was due to water evaporation of the samples in the first step. In contrast, the successive weight loss between 200 and 400 °C could be attributed to the thermal decomposition of the samples. After the heating procedure, the TGA curve of CMC began to decrease gradually, and some functional groups degraded. The curve was then progressively maintained at the same level from 70 to 240 °C, after which it dropped sharply to lose approximately 50% of its weight at 320 °C, leaving a 40% residual weight^54^.

In contrast, the TGA curve of GO was significantly thermally unstable, with over 55% of its weight loss occurring below 150 °C. GO was decomposed in four stages: moisture evaporation at 39–96 °C, dehydration of adsorbed water at 96–152 °C, pyrolysis of O-rich functional groups –OH and C–O–C at 152–271 °C, and –COOH decomposition at 271–379 °C. At 800 °C, GO loses approximately 80% of its mass^55^. It was also discovered that combining CMC and GO in a composite improved both, indicating that GO and CMC nanoparticles interact strongly. The CMC-GA-GO_102_ curve began to decrease at 120 °C and then exhibited a modest change in slope until 350 °C, indicating that the degradation process of the complex composites was complete.

In addition, the residual content of CMC-GA-GO_102_ was 27%, the residual value of CMC was 16%, and the residual amount of GOs was 7%, proving that CMC was effectively modified onto the surfaces of CMC-GA-GO_102_^56^. As depicted in Fig. 4, the thermal stability of CMC-GA-GO_102_ following adsorption of the MB dye species was approximately 60%. This phenomenon may be explained by the fact that the adsorption process consumes various function groups, thereby weakening the electrostatic attraction between the precursors of the beads^57^.

### Effect of the biocomposites GO:CMC composition on the dye removal

**Figure 5:**
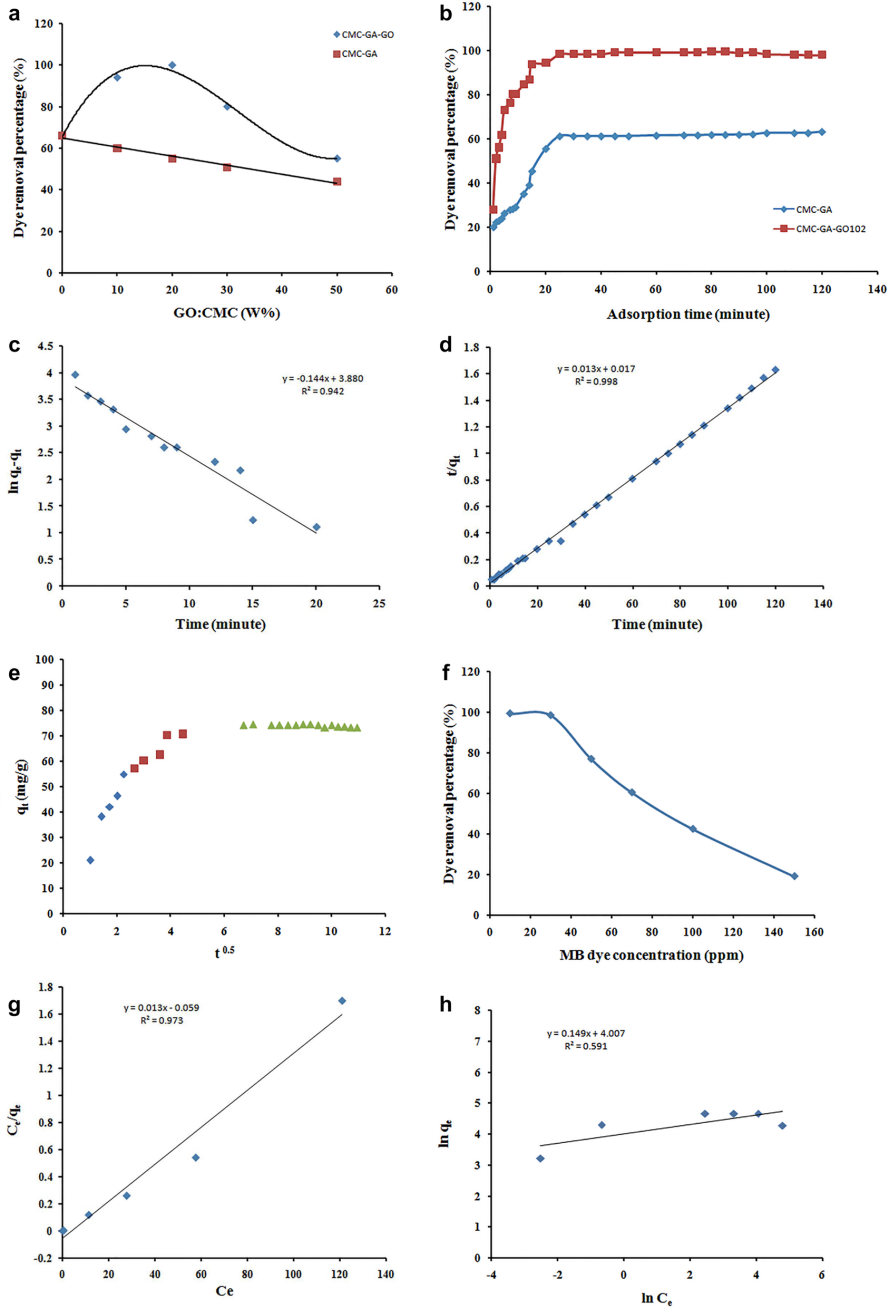

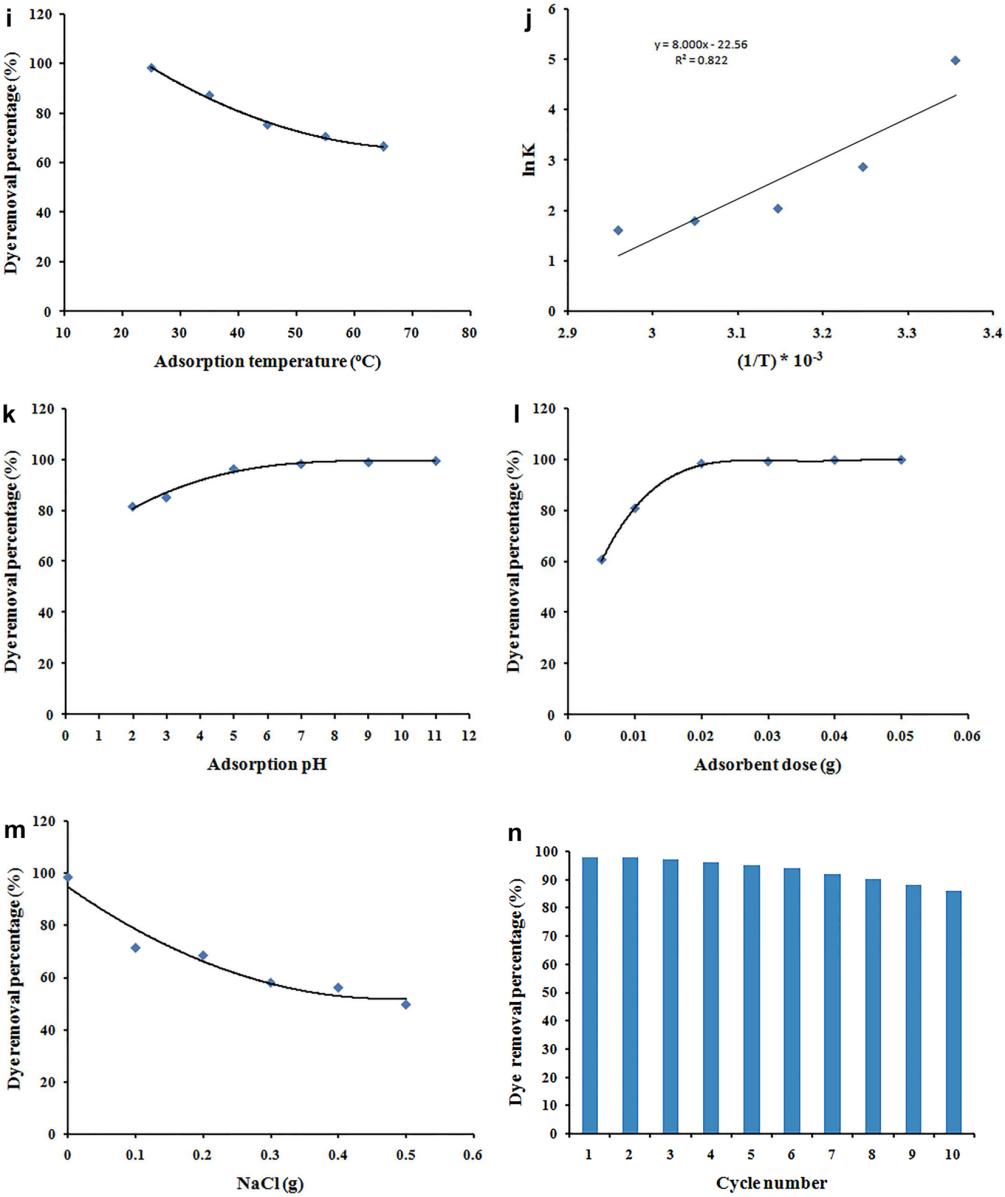
(**A**) Effect of the GO:CMC (W%) ratio of different biocomposites adsorbents on the MB dye removal percentages (%); (adsorbent dose 20 mg, adsorption temperature 25 °C, adsorption time 120 min, adsorption pH 7.0, and Dye concentration 30 ppm). (**B**) Effect of contact time on removal percent, for adsorption of MB dye onto CMC-GA and CMC-GA-GO_102_ adsorbents; (dye = 30 mg/L, dose = 20 mg, pH = 7, T = 25 °C). (**C**) The pseudo first-order model adsorption kinetics model. (**D**) The pseudo second-order model adsorption kinetics model. (**E**) The intra-partical diffusion kinetic model. (**F**) Effect of initial MB dye concentration on adsorption percent onto CMC-GA-GO_102_ adsorbent; (t = 25 min, dose = 20 mg, pH = 7, T = 25 °C). (**G**) Langmuir isotherm model. (**H**) Freundlich isotherm model. (**I**) Effect of temperature on the removal percentage of MB dye onto CMC-GA-GO_102_ adsorbent (a), and thermodynamic isotherm for the adsorption processes (b); (t = 25 min, [MB] = 30 mg/L, dose = 20 mg, pH = 7). (**J**) Van't Hoff thermodynamic isotherm model. (**K**) Effect of adsorption pH on the removal percent of MB dye from aqueous solution using CMC-GA-GO_102_; (t = 25 min, [MB] = 30 mg/L, dose = 20 mg, T = 25 °C). (**L**) Effect of CMC-GA-GO_102_ adsorbent dose on the MB removal percentage; (t = 25 min, [MB] = 30 mg/L, pH = 7, T = 25 °C). (**M**) Effect of NaCl dose on the removal percent of MB dye from aqueous solution using CMC-GA-GO_102_; (t = 25 min, [MB] = 30 mg/L, dose = 20 mg, pH = 7, T = 25 °C). (**N**) Reusability of CMC-GA-GO_102_ adsorbent in the removal of MB.

Different amounts of GO were physically mixed with CMC solution, chemically crosslinked with GA, and then freeze-dried to produce CMC-GA-GO_x_ biocomposites with a distinct porosity. The CMC-GA-GO_x_ biocomposites (Table 1) were evaluated for their ability to remove MB dye under fixed adsorption and compared the dye removal percentages (%) with the CMC-GA hydrogel-based adsorbent (without GO) to show the contribution of GO incorporation and the resulted synergetic effect (Fig. 5A).

Figure 5A depicts the percentage of MB dye removed by the CMC-GA-GO_x_ biocomposites, which is generally more significant than the CMC-GA counterpart. On the other hand, the GO:CMC (W%) ratio has a distinct positive synergetic effect on the dye removal percentage, with GO:CMC (20%) providing the best results. Further, an increase in the GO weight ratio in the biocomposites adsorbents reduces positive synergetic effect on the percentage of dye removal until it reaches the minimum increment value of the CMC-GA-GO_x_ adsorbent with a 50% GO:CMC weight ratio. The data presented in Fig. 5A indicate that the positive synergetic effect on the dye removal percentage increment were found 56.66%, 81.8%, 57.6%, and 25% for the CMC-GA-GO_101,_ CMC-GA-GO_102,_ CMC-GA-GO_103,_ CMC-GA-GO_105_, respectively.

The explanation of the positive synergetic effect of GO incorporation could be referred to one or more of the following;

- The high surface area and consequently high number of adsorption active sites of GO compared with CMC,
- Improvement of the composite porosity,
- Creating of driving force through the concentration gradient between the MB aqueous phase and the adsorbent solid one,
- Blocking of the pores with agglomerated GO particles.

The compromise between the above mentioned factors leads to obtained results which the CMC-GA-GO_102_ biocomposite has the highest increment percentage of MB dye removal compared with its CMC-GA counterpart which was almost doubled.

Increasing the incorporated GO above 20% leads to reducing the composite porosity as a result of blocking the pores with the agglomerated GO particles which creates a diffusion barrier of the MB from the aqueous phase to the adsorbent solid phase. Above all, the concentration gradient greatly reduced as a result of increased the GO adsorption active sites and consequently fast equilibrium was reached. Accordingly, the CMC-GA-GO_102_ biocomposite was utilized to study the adsorption processes' operational conditions and evaluate the kinetic, isotherm, and thermodynamic properties of the MB adsorption process.

### Effect of contact time and adsorption kinetics

Figure 5B depicts the effect of contact duration on the adsorption of MB dye by CMC-GA and CMC-GA-GO_102_.CMC-GA-GO_102_ eliminated 98.3% of MB dye, while CMC-GA eliminated 61.25%. The adsorption removal and adsorption capacities of CMC-GA-GO_102_ for MB dye increased significantly within the first 25 min. A large number of active sites on the CMC-GA-GO_102_ sorbent surface were capable of producing significant removal activity. The adsorption rate decreases due to the gradual filling of the adsorption sites. There is insufficient contact between CMC-GA-GO_102_ and MB dye species for the first 25 min, but equilibrium is progressively reached after sufficient contact. This behavior is a result of the dye molecules occupying the active sites and the adsorbent appearing saturated with the pollutant^33^, on the one hand, and the concentration gradient between the nano-biocomposite adsorbent solid phase and the MB dye solution phase, which is primarily responsible for facilitating the transfer of the MB molecules from the solution to the adsorbent phase, decreasing over time until equilibrium is reached, on the other hand. The equilibrium adsorption capacity was determined to be 73.73 mg/g.

Kinetic studies for the adsorption of various pollutants, such as synthetic dyes and heavy metal ions, are essential because they provide information on the time required to reach adsorption equilibrium, the rate of adsorption, and the concentration of adsorbate in each phase after equilibrium has been reached^58^.

To better comprehend the adsorption of MB dye onto CMC-GA-GO_102_ composite, the kinetic behavior was studied using Pseudo-first order (PFO) and Pseudo-second order (PSO) as well as Intra-particle diffusion (IPD) kinetics models, which can be expressed as follows. The linear equation for the model of pseudo-first order is^36^;

3 $$\mathrm{ln}({q}_{e}-{q}_{t})=\mathrm{ln}{q}_{e}-{K}_{1}t$$

where q_e_ is the adsorption amount of dye in mg/g at equilibrium**,** q_t_ (mg/g) is the Amount of MB dye adsorbed at time t and K_1_ (min^−1^) is the first order kinetic model’s rate constant.

Table 1 summaries the values of K_1_, q_e_, and the correlation coefficient (R^2^) calculated from linear plots of $\mathrm{ln}(\mathrm{qe}-\mathrm{qt})$ versus t (Fig. 5C).

The linear equation of the PSO model is^59^;

4 $$\frac{t}{{q}_{t}}=\frac{1}{{k}_{2}{{q}_{e}^{2}}}+\frac{t}{{q}_{e}}$$

K_2_ is the rate constant of pseudo second-order kinetic model's adsorption (Fig. 5D).

The linear equation for Intra-partical diffusion model is^60^;

5 $$qt={k}_{p}{t}^{0.5}+c$$

K_p_ is the rate constant of the intra-particle diffusion kinetic model's (mg g^−1^/min) and it^’^s value can be found from the slope of qt versus t^0.5^ and C (mg g^−1^) is the intercept gives an indication of the boundary-layer thickness (Fig. 5E).

The parameters calculated from the slope and intercept of the linear plots of the Pseudo-First order, Pseudo-Second order, and Intra-particle diffusion models are shown in Fig. 5C–E and tabulated in Table 2.

**Table 2:** Adsorption kinetics and diffusion mechanism for removal of MB dye.

q_e-exp_ (mg/g)	First-order kinetic parameter (PFO)			Second-order kinetic parameter (PSO)		
	K_1_ (min^−1^)	q_e-cal_ (mg/g)	R^2^	K_2_ (g/mg min)	q_e-cal_ (mg/g)	R
73.73	− 0.144	48.42	0.94	0.017	76.92	0.998

Intra-particle diffusion (IPD)						
Adsorption			Saturation			
C	K	R^2^	C	K	R^2^	
− 1.405	25.0	0.95	76.39	− 0.252	0.543	

Table 2 demonstrates that R^2^ of the Pseudo-second order model (R^2^ = 0.998) is greater than R^2^ of the Pseudo-First order model (R^2^ = 0.942) for MB dye. Moreover, the calculated equilibrium capacity values (q_e-cal_ = 76.92 mg/g) form the Pseudo-second order were found to be more closer to the corresponding experimental values (q_e-exp_ = 73.73) than those of Pseudo-First order model (q_e-cal_ = 48.42 mg/g), indicating that the Pseudo-second order kinetic model can be well explain the kinetic of the adsorption of MB dye onto the surface of CMC-GA-GO_102_ composite. The three-stage adsorption of MB dye on the surface of CMC-GA-GO_102_ composite was disclosed by plotting q_t_ versus t^0.5^ (Fig. 5E). The first stage may be attributed to the diffusion of MB molecules from the bulk to the exterior surface of the CMC-GA-GO_102_ composite. The second stage consists of the MB dye molecules' delayed diffusion. In the final stage, equilibrium is declared to have been attained. The linear plot of the intra-particle model (Table 2) revealed that the straight line has nonzero intercept values, indicating that the adsorption of MB dye on CMC-GA-GO_102_ is likely to be complex and involve both film diffusion (boundary layer diffusion) and intra-particle diffusion^61^.

### Effect of initial dye concentration and adsorption isotherms

The eradication of dye is highly dependent on the initial concentration of dye. The effect of initial dye concentration is determined by the direct relationship between the number of dye molecules in the solution and the available number of active sites on an adsorbent. Figure 5F depicts the effect of initial dye concentrations (10–150 mg/L) on the adsorption percentages (%R) of MB dye from aqueous solution onto CMC-GA-GO_102_ nanocomposite was investigated. At the same time, all other parameters were held constant. Figure 5F demonstrates that the adsorption percentage of MB dye onto CMC-GA-GO_102_ decreases as the initial concentration of MB dye increases at 25 min of adsorption time. This effect is because the adsorbent had many active sites for the adsorption of the dye species at low initial dye concentrations. When the dye concentration was increased, these vacant sites became saturated with MB dye species and lost their ability to absorb more dye molecules^62–64^. Consequently, as dye concentrations increase, adsorption efficiency decreases^65^.

Two adsorption isotherm models (Langmuir and Freundlich) were used to investigate the adsorption performance of the CMC-GA-GO_102_ composite and the interactions with the MB dye molecules^66^.

The Langmuir model assumes that a single layer of adsorption forms a homogeneous surface to explain the adsorption of MB dye on the surface of CMC-GA-GO_102_ based on the correlation coefficient (R^2^ = 0.973), which indicates that the adsorption process occurs on a homogeneous active site as a monolayer; Fig. 5G. Its equation is given in^67^.

6 $$\frac{{c}_{e}}{{q}_{e}}=\frac{1}{{k}_{l}{q}_{m}}+\frac{{c}_{e}}{{q}_{m}}$$

where q_e_ (mg/g) is the amount of MB dye adsorbed onto the surface of the CMC-GA-GO_102_ composite at equilibrium. C_e_ (mg L^−1^) is the MB equilibrium concentration. q_m_ (mg/g) represents the maximum adsorption capacity of MB dye onto the adsorbent surface and K_L_ (L/mg) represents the Langmuir constant. The slope and intercept of the C_e_/q_e_ against C_e_ plot were used to calculate the values of q_m_ and K_L_.

In contrast, the Freundlich model proposes the existence of a heterogeneous adsorbent surface^68^ is represented by the following equation:

7 $$\mathrm{ln}{q}_{e}= {k}_{f}+\frac{1}{n}\mathrm{ ln}{c}_{e}$$

k_F_ (mg/g) and n represent Freundlich constants which describe adsorption capacity and strength, respectively, and can be obtained from the intercept and slope of plotting ln q_e_ with ln C_e_; Fig. 5H. Furthermore, the 1/n values for MB dye is 0.149, indicating that the procedure is not conducive to adsorption^69^. The related parameters calculated from the adsorption isotherm of MB dye species adsorption by CMC-GA-GO_102_ are listed in Table 3. R^2^ = 0.973 for MB indicates that the adsorption of MB dye onto the CMC-GA-GO_102_ composite surface is homogeneous, as predicted by the Langmuir model. However, MB's Freundlich isotherm determination coefficient (R^2^) was 0.591. The determination coefficient (R^2^) of the Langmuir isotherm was much higher than that of the Freundlich isotherm, the theoretical q_m_ obtained from the Langmuir model was found to be 76.92 mg/g for MB matching the experimentally determined value (73.73 mg/g), while the theoretical q_m_ obtained from the Freundlich isotherm was found to be 56.347689 mg/g. The obtained results confirmed the complete homogenization of the GO nanoparticles in the CMC-GA solution.

**Table 3:** Isotherms constants for MB adsorption onto CMC-GA-GO_102_ adsorbent.

Qe _exp_ (mg/g)	Langmuir isotherm model			Freundlich isotherm model		
	Q_max_ (mg/g)	K_L_ (L/mg)	R^2^	η_f_	K_f_ (mg/g)	R^2^
73.73	76.92	4.54	0.973	6.71	55	0.591

### Effect of adsorption temperature and thermodynamics

Figure 5I illustrates the effect of dye solution temperature in the range of (25–65 °C) on the adsorption efficiency of MB onto CMC-GA-GO_102_ adsorbent with all other factors held constant. When the aqueous solution temperature increased from 25 °C to 65 °C, the MB dye adsorption efficiency decreased from 98.3 to 66.5%, indicating that MB dye adsorption onto CMC GA-GO_102_ composite is an exothermic process. Therefore, to conserve energy, we chose 25 °C for the subsequent series of investigations^70^.

The decrease in adsorption efficiency can be explained by an increase in the propensity for the adsorbed dye to dissociate from the solid surface and leach into the liquid, the contraction of active sites at high working temperatures, and a decrease in the effective concentration gradient driving force^12^. That is likely because the thermal motion of the MB molecules accelerated at high temperatures, resulting in a rapid equilibrium between adsorption and desorption^36^. Therefore, thermodynamic studies were used to investigate the thermal changes and spontaneous ability of MB dye's adsorption processes and reactions system onto CMC-GA-GO_102_ composite (Fig. 5J).

The standard Gibbs free energy change (G), the standard enthalpy change (H), and the standard entropy change (S) were measured as thermodynamic parameters using Van't Hoff equations^71,72^ to learn more about the nature of this process.

8 $$\mathrm{ln}\frac{{q}_{e}}{{c}_{e}}=\frac{\Delta S}{R}-\frac{\Delta H}{RT}$$

9 $$K=\frac{{q}_{e}}{{c}_{e} }$$

10 $$\Delta G=-\mathrm{RTln}K $$

*K* is the thermodynamic equilibrium constant, T (K) is the temperature, and R (8.314 J mol^−1^ K^−1^) is the general gas constant. H/R is calculated according to Eq. (8) by plotting ln K versus 1/T. The y-axis intercept is used to calculate S/R. Table 4 displays the parameters for the adsorption of MB dye onto CMC-GA-GO_102_ composite derived from thermodynamic calculations measured by the linear relation of van't Hoff equation at various temperatures. The decreasing K values with increasing temperature and the negative H° value indicate that MB adsorption onto the CMC-GA-GO_102_ composite is exothermic. In addition, the entropy (S) change was negative (-187.56 J/mol K), indicating that the process is more practicable at lower temperatures. In addition, the decrease in negative G° values with increasing temperature indicates that the process is more spontaneous at lower temperatures.

**Table 4:** **C**alculated Thermodynamic parameters for the sorption of MB onto CMC-GA-GO_102_.

ΔH° (J/mole)	ΔS° (J/mole K^−1^)	ΔG° (J/mol)				
		298 k	308 k	318 k	328 k	338 k
− 66.51	− 187.56	− 12,328.40	− 7328.76	− 5385.53	− 4878.59	− 4507.45

### Effect of adsorption pH

The treatment pH is one of the most influential factors on adsorbent capacity in wastewater treatment, as it substantially affects the adsorbent surface charge, dissociation of the functional groups of the adsorbents, and the degree of ionization of the adsorptive species consequently, removal effectiveness. Therefore, pH is a factor that controls adsorption.

The effect of solution pH on CMC-GA-GO_102_ removal efficiency was studied by modulating the pH of MB dye solution from 2 (extremely acidic) to 11 (extremely alkaline) while maintaining all other parameters constant. The synthesized anionic CMC-GA-GO_102_ nanocomposites, including the hydroxyl and carboxyl functional groups, were affected by the pH of the dye solution. Figure 5K illustrates how pH affects the adsorption of MB dye species. It was evident that the removal (%) for MB increased from 81 to 98.3% as the pH rose from 2 to 11. The lower efficiency at low pH values (strongly acidic) was due to the carboxyl and hydroxyl functional groups are mostly in non-ionized (–OH, –COOH) form and a low interaction can occur between the cationic dyes and these groups^6^; also the H^+^ ions existing in the solution compete with the cationic MB for negative sites of the adsorbents like carboxylic acid. Thus, it is difficult for MB molecules to diffuse on the adsorbent surface^73^; hence, the dye removal percentage (%) is reduced. The adsorption capacity increased linearly with the increase of pH value, up to pH 7. At this pH value, the carboxyl and hydroxyl functional groups of the CMC-GA-GO_102_ nanocomposites dissociate. Therefore, these groups are present in –O^−^ and –COO^−^ form in the CMC-GA-GO_102_ nanocomposites, and strong electrostatic interactions could occur between the positive site of MB and the negative site of the CMC-GA-GO_102_ adsorbent. Likewise, at pH values higher than 7, up to pH 11, there was a lower rate of increase in the adsorption that could be related to the existence of a large amount of Na^+^in the MB solution medium reduced interactions between MB and the anionic functional group of CMC-GA-GO_102_ adsorbent^73,74^.

### Effect of the CMC-GA-GO_102_ dosage

Figure 5L depicts the percentages of dye adsorption (R%) at different CMC-GA-GO_102_ dosage (mg). The results demonstrated that, at constant MB dye concentration, the adsorption percentage of MB increases from 60.5 to 99.8% with increasing CMC-GA-GO_102_ adsorbent dose in the range of 0.005–0.05 g, which directly increased the number of active sites, thereby increasing the adsorption efficiency^19,75,76^. However, the dye removal percentage is nearly constant when mixing 0.02 g of CMC-GA-GO_102_ composite, so that the current investigation will use 0.02 g of CMC-GA-GO_102_ adsorbent.

### Effect of NaCl dose

Under optimal conditions, the effect of NaCl on the removal percentages of MB dye using CMC-GA-GO_102_ nanocomposite was evaluated and illustrated in Fig. 5M.

As shown in the figure, varying concentrations of sodium chloride in the range of 0.1 to 0.5 g were used to determine the influence of salt concentration on the %R of the investigated dye on CMC-GA-GO_102_. The removal percentage of MB dye decreased swiftly after the addition of NaCl. Electrostatic filtration effects are the primary cause of this effect. In other terms, Na^+^ ions and cationic MB molecules compete for anionic binding sites on the CMC-GA-GO_102_ adsorbent. As a result, their absorption is diminished. By enhancing the ionic strength of the aqueous medium, the presence of NaCl shields the active surface sites. Positively charged Na ions in aqueous dye solutions may decrease the negative charge of active sites on the adsorbent surface, thereby diminishing the electrostatic attraction forces between dye molecules and negatively charged active sites. Moreover, the presence of NaCl in the dye solution phase creates an osmosis difference that causes water molecules to migrate from the swollen CMC-GA-GO_102_ adsorbent to the MB liquid phase, resulting in de-swelling of the CMC-GA-GO_102_ adsorbent and a diffusion barrier against the MB molecules entering the adsorbent's interior porous structure. By illustration, the system containing no added salt had an MB adsorption percentage of approximately 98.3%, whereas the system containing 0.5 g NaCl had a value of approximately 49.6%^21,59^.

### Reusability

The main goal of regeneration process is to reuse the adsorbent materials for many times as much as possible. Cycles of adsorption and desorption have been tried under the same conditions using 3% NaCl solution for the CMC-GA-GO_102_-MB regeneration (Fig. 5N). It is clear from the figure that gradual decreases of the MB dye removal percentage has been observed with successive adsorption–desorption cycles. However, the MB dye removal percentage reaches 86% after ten adsorption–desorption successive cycles. That result indicates that the CMC-GA-GO_102_ adsorbent lost only 14% of its adsorption efficiency and consequently proved the capability of reusing the developed CMC-GA-GO_102_ adsorbent.

### Comparative study

Table 5 presents a comparative analysis of MB removal by chemically modified polyacrylonitrile. Additionally, additional absorbents are included. It is evident from the table that Salisu et al.^77^ eliminated methylene blue (MB) dye with alginate graft-polyacrylonitrile beads. They discovered that the maximal monolayer coverage of Langmuir was 3.51 mg/g. Kiani et al.^78^ developed polyacrylonitrile (PAN) and monoethanolamine-based chelating resins to remove methylene blue from an aqueous solution. The maximal adsorption capacity was determined to be 52.3 mg/g. Abu-Saied et al. The Langmuir adsorption capacity (Q_o_) was determined to be 54 mg/g. Natural adsorbents, such as H. cannabinus-g-PAA and H. cannabinus-g-PAA/PAAM, have a limited adsorption capacity (7.0 mg/g)^80^, regardless of grafting with functional polymers. The adsorption capacity of activated lignin-chitosan composites and brown macroalga is moderately moderate (35–36 mg/g)^81,82^. The adsorption capacity of inorganic adsorbents such as MOF is moderately high (326 mg/g)^83^. The maximum adsorption capacity of nano-hydroxamate polyacrylonitrile (HPAN) particles is 8.34 (mg/g)^84^. The CMC-GA-GO_102_ adsorbent developed in the present study has a monolayer adsorption capacity of 106.95 mg/g.

**Table 5:** Comparison of the MB adsorption capacity of different adsorbents.

Adsorbent matrices	Capacity (mg/g)	MB dye removal conditions	References
PAN-g-Alginate	3.51	6 ppm, pH 7.0, 40 min, R.T, and 0.1 g adsorbent	^77^
PAN-g-monoethanolamine	52.3	50 ppm, pH 9.5, 100 min, R.T, and 0.1 g adsorbent	^78^
Iminated polyacrylonitrile (IPAN)	54	800 ppm, pH 7, 3 h, 40 °C, and 0.3 g adsorbent	^79^
MOF	326	400 ppm, pH 8.9, 2 h, 40 °C, and 0.02 g adsorbent	^83^
H. cannabinus-g-PAA	7.11	11 ppm, pH 7, 2 h, 25 °C, and 0.03 g adsorbent	^80^
H. cannabinus-g-PAA/PAAM	7.00	11 ppm, pH 7, 2 h, 25 °C, and 0.03 g adsorbent	^80^
Activated liginin-chitosan Blends	36.25	80 ppm, pH 7, 40 h, 20 °C, and 0.05 g adsorbent	^81^
Brown macroalga	35	200 ppm, pH 6.5, 2 h, 27 °C, and 1.5 g adsorbent	^82^
HPAN	8.34	50 ppm, pH 7, 3 h, 25 °C, and 0.2 g adsorbent	^84^
CMC-GA-GO_102_	76.92	150 ppm, pH 7, 25 min, 25 °C, 20 mg adsorbent	This work

## Conclusion

Glutaraldehyde crosslinked sodium carboxymethyl cellulose (CMC-GA) hydrogel, and its nanographene oxide composite (CMC-GA-GO_x_) were used to develop an effective carboxymethyl cellulose-graphene oxide biobased composites adsorbent for the removal of methylene blue (MB) cationic dye contaminate from industrial wastewater. The optimal GO weight percentage was discovered to be 20%; CMC-GA-GO_102_. Several variables affecting the elimination of MB dye have been investigated. After 25 min of adsorption, 98% of the 30 ppm MB was removed, indicating that the equilibrium was reached rapidly. The optimal pH range for adsorption was between 2.0 and 5.0, where the removal (%) increased from 81.0 to 93.0% for MB, whereas the removal percentage reached equilibrium at 98.0% with a pH of 11. When the aqueous solution temperature increased from 25 to 65 °C, the MB dye adsorption efficiency decreased from 98.3 to 66.5%, indicating that MB dye adsorption onto CMC GA-GO_102_ composite is an exothermic process.

Regarding initial MB concentration, the adsorption %R of MB dye onto CMC-GA-GO_102_ decreased almost linearly from 30 to 150 ppm at 25 min of adsorption time. The highest MB removal percentages were obtained within a concentration range of 10 to 30 ppm. At constant MB dye concentration, the adsorption percentage of MB increases from 60.5 to 98.0% with increasing CMC-GA-GO_102_ adsorbent dose in the range of 0.005–0.02 g, where equilibrium was nearly attained. Increasing the adsorbent dose to 0.05 g resulted in a modest increase in the MB removal percentage. Any concentration of NaCl harms the MB removal percentage. The experimental data of the adsorption process were more consistent with the Langmuir isotherm, in which the maximal monolayer adsorption capacity was determined to be 76.92 mg/g. The adsorption process followed the kinetic model of pseudo-second order. The removal of MB was exothermic and spontaneous from a thermodynamic standpoint. In addition, thermodynamic results demonstrated that adsorption operates most effectively at low temperatures. These results suggest that the CMC-GA-GO_102_ composites could be a cost-effective adsorbent for removing organic cationic dyes from industrial wastewater. Cycles of adsorption and desorption have been tried under the same conditions using 3% NaCl solution for the CMC-GA-GO_102_-MB regeneration. The MB dye removal percentage reaches 86% after ten adsorption–desorption successive cycles. That result indicates that the CMC-GA-GO_102_ adsorbent lost only 14% of its adsorption efficiency and consequently proved the capability of reusing the developed CMC-GA-GO_102_ adsorbent. A comparative analysis of the removal of MB by various other adsorbents revealed that the CMC-GA-GO_102_ adsorbent developed in this study has a moderately high monolayer adsorption capacity.

In addition, the CMC-GA-GO_102_ composite was characterized using FTIR, RAMAN, TGA, SEM, and EDX analysis instruments. As a result of the interaction between the CMC-GA-GO_102_ adsorbent and the MB dye molecules, the FTIR spectrum displays peaks corresponding to the dye species. After the adsorption processes of dye species, observations of shifts, disappearances, the emergence of new bands, and alterations in peaks were observed. At 1215 cm^−1^, 879 cm^−1^, and 832 cm^−1^, distinctive peaks appeared for C–N stretching, aromatic C–H bond, and out-of-plane bending, respectively. The peak at 3362 cm^−1^ became 3434 cm^−1^, the peak at 1636 cm^−1^ became 1588 cm^−1^, the peak at 1319 cm^−1^ became 1391 cm^−1^, and the peak at 1268 cm^−1^ became 1215 cm^−1^. After surface adsorption of MB dye, the intensity and location of peaks in the structure of CMC-GA-GO_102_ nanocomposite changed. It signifies a reaction between the adsorbent and the contaminant dye. In addition, the EDX analysis of the CMC-GA-GO_102_-MB reveals that it contains carbon, oxygen, nitrogen, sulfur, and chlorine. Furthermore, the Na and O content of the CMC-GA-GO_102_ will decrease, proving the MB dye adsorption.
